# Indoor augmented reality (AR) pedestrian navigation for emergency evacuation based on BIM and GIS

**DOI:** 10.1016/j.heliyon.2024.e32852

**Published:** 2024-06-12

**Authors:** Mojtaba Valizadeh, Babak Ranjgar, Alessandro Niccolai, Hamid Hosseini, Soheil Rezaee, Farshad Hakimpour

**Affiliations:** a Department of Geomatics, University College of Engineering, University of Tehran, Tehran, 1439957131, Iran; b Department of Energy, Politecnico di Milano, La Masa, 34, Milan, 20156, Italy; c Faculty of Geodesy and Geomatics Engineering, K. N. Toosi University of Technology, Tehran, 1996715433, Iran

**Keywords:** Augmented reality (AR), BIM, Emergency evacuation, GIS, Indoor navigation, Indoor positioning

## Abstract

Nowadays with the increase of high-rise buildings, emergency evacuation is an indispensable part of urban environment management. Due to various disaster incidents occurred in indoor environments, research has concentrated on ways to deal with the different difficulties of indoor emergency evacuation. Although global navigation satellite systems (GNSSs) such as global positioning system (GPS) come in handy in outdoor spaces, they are not of much use in enclosed places, where satellite signals cannot penetrate easily. Therefore, other approaches must be considered for pedestrian navigation to cope with the indoor positioning problem. Another problem in such environments is the information of the building indoor space. The majority of the studies has used prepared maps of the building, which limits their methodology to that specific study area. However, in this study we have proposed an end-to-end method that takes advantage of BIM model of the building, thereby applicable to every structure that has an equivalent building information model (BIM). Moreover, we have used a mixture of Wi-Fi fingerprinting and pedestrian dead reckoning (PDR) method with relatively higher accuracy compared to other similar methods for navigating the user to the exit point. For implementing PDR, we used the sensors in smartphones to calculate user steps and direction. In addition, the navigational information was superimposed on the smartphone screen using augmented reality (AR) technology, thus communicating the direction information in a user-friendly manner. Finally, the AR mobile emergency evacuation application developed was assessed with a sample audience. After an experience with the app, they filled out a questionnaire which was designed in the system usability scale test (SUS) format. The evaluation results showed that the app achieved an acceptable suitability for usage.

## 1 Introduction

Emergency evacuation is a key risk-reduction strategy for disaster-threatened buildings, thus a necessary part of urban environment management and safety [1]. There has been extensive study on simulation and planning of population evacuation in times of disasters such as earthquake, inundation, and hurricane [[2], [3], [4]]. Recently, there has been a focus on emergency evacuation in indoor environments following catastrophic incidents causing many casualties and economic losses [[5], [6], [7]]. Especially the tragic fire-induced collapse of the 16-story Plasco building in Tehran, Iran in 2017 [8] was the main motivation of this study. In this shocking incident the entire steel-structured commercial building collapsed after being on fire for about 4 h causing the death of twenty-two people of whom sixteen were firefighters. Humans nowadays spend most of their time indoor [9] (up to 90 %) and due to increasing complexity of buildings, an effective emergency evacuation system for occupants in times of emergency like fire is a must [10,11]. In many cases, especially in public buildings where many people do not have a thorough knowledge of the building, one of the main problems in indoor discharge is uncertainty of routes [12]. Few minutes of delay in navigation to safety exits can cause loss of lives [13].

Indoor localization and navigation is a challenge in the absence of global navigation satellite systems (GNSS) such as global positioning system (GPS). Although methods like assisted GPS (AGPS) are developed for places with limited line of sight to GPS satellites, they are not practical for indoor positioning because the multipath problem is a serious challenge in buildings and signal strength still remains too low for inner parts [14]. However, with the advent of sensors and smart phones indoor localization has become much affordable with acceptable accuracy. Based on these advanced technologies multiple approaches are developed and used for indoor navigation such as Bluetooth [15], RFID [16], Wi-Fi-based techniques [17], and pedestrian dead reckoning (PDR) methods using inertial measuring unit (IMU) [18]. There are various indoor positioning and navigation methods and technologies developed and it is continuously evolving. For a better understanding of this subject and different classifications, it is suggested to refer to the following studies [[19], [20], [21], [22]].

Information about indoor spaces could be very helpful for different applications such as indoor positioning and navigation, indoor air quality monitoring, 3D cadaster, etc. which can be obtained by accurate 3D building models. Building information modeling (BIM) is an intelligent model-based process that can be used to create and manage information required for architecture, engineering and construction (AEC) projects [[23], [24], [25]]. In fact, BIM models are integrated databases that can be shared among stakeholders in order to increase efficiency of collaboration throughout entire facilities life cycle including planning, design, construction, operation and maintenance [25]. As a model, BIM represent physical and functional information about a facility such as building and provide very detailed 3D geometric and semantic information mostly for indoor applications. For example, in Ref. [26], a new indoor map model, which is named building information modeling, based positioning and navigation (BIMPN) is developed which is based on connecting entity models and network models. In the network model, which is for creating a 3D indoor path network, rooms, facilities, doors and windows are considered as horizontal nodes and stairs and elevators consist vertical nodes. These nodes are connected to each other by corridors and connect relations. On the other hand, in the entity model, which is for improving the network visualization inside the building, the walls, columns, floors, doors, windows are considered as horizontal elements, and stairs and elevators are considered as vertical elements. From Geo-informatics view, BIM can be considered as a shared storage for rich and detailed spatial indoor information [27,28]. However, BIM lacks convenience tools for different spatial analysis like path finding. In this regard, integration of Geospatial Information Systems (GIS) and BIM could increase the efficiency of indoor information management by adopting its spatial tools for different purposes [24,29].

In augmented reality (AR) systems, information and graphics such as virtual objects, multimedia, and text are superimposed in the real-world view of users to provide instant additional information for better interaction users with the environment [30]. Today, people can easily access AR systems in their smartphones, an example of such applications is Pokémon Go. AR systems are employed in variety of fields including education [31], gaming [32], tour guides [33], infrastructure maintenance [34], recommender systems [35], and recently AR is used in indoor emergency evacuation systems for its better user experience and fast information communication to users [36]. Moreover, AR can facilitate training of occupants and simulation of various hazards in indoor environments [37]. However, there are some concerns regarding AR systems that must be addressed. Lovreglio and Kinateder have done a comprehensive SWOT (strengths, weaknesses, opportunities, and threats) analysis on the application of AR systems for pedestrian evacuation [38].

In the next section a concise and focused literature review on AR indoor pedestrian evacuation is presented. Then, an overall process of the system is presented. Next, in the fourth section, implementation of various aspects of the proposed approach such as indoor positioning, navigation methodology, AR interface and the process of path generation and visualization on users’ view of real world is presented. Moreover, Section five is dedicated to the evaluation of the system. Finally, the last section concludes the work and discusses limitations and potential areas of improvement.

## 2 Literature review

The number of papers on the subject of AR for pedestrian navigation is not large compared to other topics. Moreover, few of these studies have been done regarding indoor emergency evacuation [38] and therefore, in this paper, we are going to develop a mobile augmented reality application that navigates pedestrians to safe exists in times of emergency. Inoue et al. [39], developed an indoor emergency evacuation system that detects fire incidents in a building using sensors located in the environment in the first phase and then alerts users and autonomously navigates them to safe exists. The system uses signals from beacons placed in the building for localization of users and generates exit paths within a 2D map on users' terminals and when the user reaches to a certain destination, the system displays photos of the place and graphical guidance. Manas et al. [40], developed AVANTI, a mobile AR navigation system using Wi-Fi for indoor positioning and accelerometer sensors for predicting speed and motion. The system was used for encouraging users for engaging in fire escape drills. Ahn and Han [41] proposed RescueMe mobile application for emergency evacuation, incorporating PDR and AR. They used marker less target detection for user localization and utilized built-in sensors of mobile to establish personalized pedometry. Ortakci et al. [42], proposed an AR evacuation system that uses RFID tags for user localization and navigates user in prepared graph of the building comprising nodes for each section of the building and lines for its corridors. They used artificial neural network (ANN) for generating a personalized escape path based on users’ different features. In another recent study, Depari et al. [43], proposed a comprehensive evacuation monitoring and management system for workers in office or factory buildings. The system is composed of two apps, one for navigation of workers to exits and another for guiding rescuers to trapped victims and critical points and a central management unit to monitor the rescue progress. The system uses received signal strength indication (RSSI) of Wi-Fi networks and also sensors located in the environment for detecting fire events. Diao and Shih [44] developed a system called MARINS, which can work in dark environments (0 lux). They employed AR within IOS using simultaneous localization and mapping (SLAM) inside a simulated area created by cardboards. Although the system shows a good performance, the 3D environment model is pre-fixed and it is difficult to adapt the system to other case studies.

As opposed to previous studies that used prepared maps of the building, we aim to take advantage of BIM model in generating geometric network of the indoor environment. Since BIM is an standard for building information modeling, it gives the system the capability to be further extended and interoperable with other systems that use this standard. Also, as BIM models are not common for buildings to have, we have proposed a procedure to develop BIM from the simple 2D CAD designs that are widely used. For initial positioning of user, Wi-Fi RSSI is used and for navigation purpose, mobile sensors are utilized to update position of pedestrians. Nowadays, most of the buildings are equipped with a network of Wi-Fi access points and almost everybody uses a smartphone containing sensors. This makes the proposed system in this study practical and applicable to majority of buildings. The navigational guide to users is provided through augmented reality for a fast and better experience of users. The aim of this study is to propose an end-to-end method that takes advantage of BIM model of the building for providing emergency evacuation paths. A mixture of Wi-Fi fingerprinting and pedestrian dead reckoning (PDR) method are used to achieve a relatively high accuracy as well as guaranteeing practicality, thus high robustness compared to other similar methods for indoor navigation. This work seeks to facilitate the emergency evacuation of buildings of which many occupants have little knowledge and have not had specific preparation for emergency evacuation.

## 3 Methodology

In the present study, our aim is to develop an indoor mobile AR emergency evacuation system by benefiting GIS analysis tools and mobile sensors in user friendly manner. In this section we underline the system design and architecture and its comprising components.

### 3.1 System architecture

**Fig. 1:**
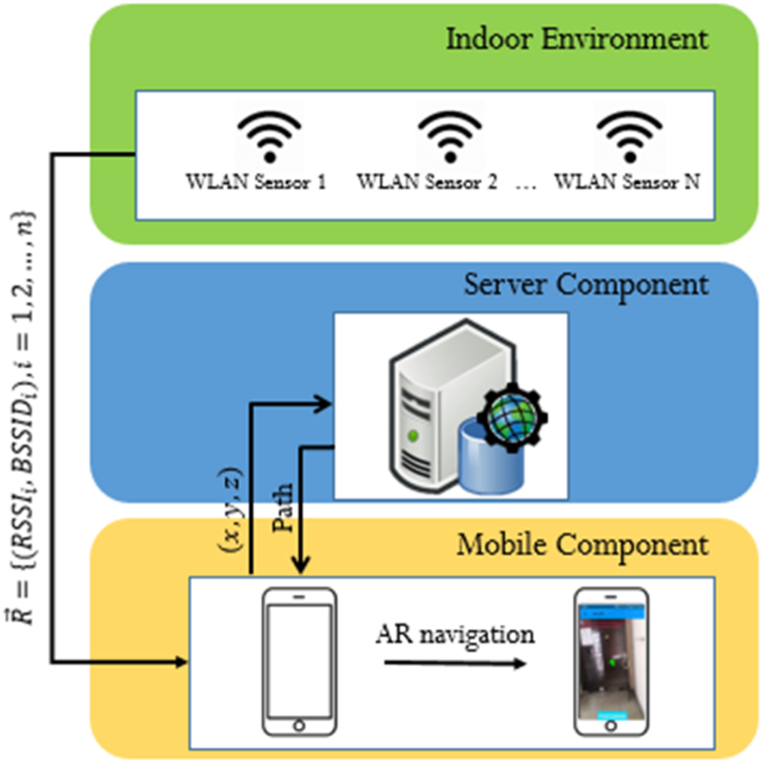
The overall system architecture.

The architecture of the proposed system is depicted in Fig. 1 and consists of three parts.

1) WLAN Sensors

The Wi-Fi sensors installed in buildings can be used for positioning in indoor environments based on the RSSI notion. In this paper, for acquiring the initial position of a pedestrian fingerprinting method based on WLAN sensors are used. This approach has several advantages; nowadays in most of the buildings these sensors are installed and used therefore, it is cost-effective. Also, the accuracy of positioning methods based on Wi-Fi sensors have proved to be provides a good compromise between availability and accuracy (2–10 m), thus suitable for initial position estimation. Furthermore, it is computationally efficient compared to other methods such as image processing and computer vision.

2) Server Component

It contains the network of the building extracted from its BIM model, published as a service, which outputs the shortest exit path by ingesting the user's initial position. Moreover, the server component has spatial analysis capabilities to calculate the shortest path from the start point that is the initial position of the user to the safe exit.

3) Mobile Component

It is the smartphone of the user than runs several steps of the process. It houses the Wi-Fi fingerprinting RSSI database that is used for calculating and allocating the initial position to the user. First, it receives the signals of the available Wi-Fi sensors and then calculates the starting point using the fingerprinting database. After that it sends the acquired initial position to the server for it to compute the exit path. Then, the mobile component receives and stores the path and using its sensor data updates the position of the user and navigates them to the next point. Finally, the mobile component runs the AR pedestrian navigation part, where the direction to the next point is graphically superimposed to the real-world view of the user for better and fast absorption of the path to the safe exist rather than the confusing fixed 2D maps and static photos.

### 3.2 Proposed approach

**Fig. 2:**
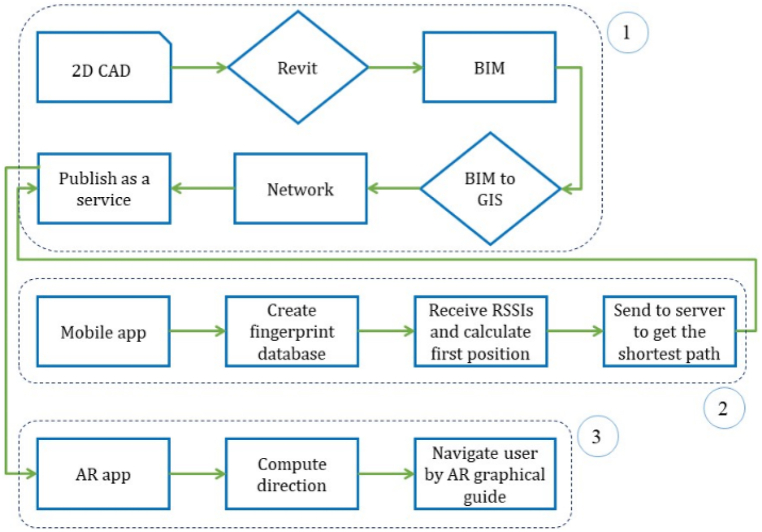
The overview of the proposed methodology.

The proposed smart mobile AR evacuation system consists of three main parts and an overview of the proposed methodology is show in Fig. 2.

1) Creating building network

In the beginning, the 2D map of the building is converted to BIM from CAD environment using Revit. Then, the BIM model is exported into a file geodatabase to be used in GIS environment for building network and spatial analysis purposes.

2) Positioning and navigation

As mentioned before, a Wi-Fi fingerprinting approach is employed for users' initial position acquisition. For this purpose, a mobile application is developed to record the RSSIs of different available WLAN sensors when standing in a specific position. With this app, the fingerprinting phase can be done easily and faster. When a user, running the AR emergency evacuation application on their smartphone, starts the app, it begins to receive the available RSSIs and using the fingerprinting database in the smartphone it can calculate the user's starting point. Next, the initial position is sent to the server component to compute the exit path.

3) Augmented Reality

Afterwards, the app receives and stores the generated path from server. Based on the initial and next positions, it then calculates the distance and direction for the user. Finally, the AR emergency evacuation app graphically represents the navigational guide on user's smartphone screen.

## 4 Implementation

In this section, various parts discussed above are presented in detail and steps taken to implement the proposed methodology are elaborated.

### 4.1 Indoor positioning and navigation

#### 4.1.1 IFC, BIM and GIS

In order to navigate inside buildings, the indoor topology of the buildings is required, which includes horizontal or vertical paths. In this regard, BIM, which includes accurate semantic and geometric information about buildings, can serve the purpose. However, as mentioned earlier, BIM models have information about building elements such as walls, columns, etc. and do not include the topological information of the indoor route network [45]. Therefore, BIM does not have the appropriate tools to build a 3D route network inside the building and perform various spatial analyses on it to perform the routing process. Consequently, extracting the information needed to create a topology from BIM models is also a challenging task. GIS can play an effective role in this regard and its integration with BIM can solve the problem of building a route network in the interior of the building more efficiently.

Various standards such as IFC [46], IDM [47] and MVD [48] are used to share BIM models and integrate them with other 3D models, such as CityGML, of which IFC is the most comprehensive standard and format for data exchange and is a common storage format in the construction industry to take advantage of the interoperability of data between different formats [49]. The IFC data model is introduced by the BuildingSMART Institute and, like other 3D models, displays 3D features, especially in buildings. This standard helps BIM models to be shared between different domains and to increase interoperability between them. One of the methods in integration of BIM and GIS is the process of extraction, transfer and loading (ETL), which converts BIM model into GIS formats semi-automatically [24]. This process extracts heterogeneous data from different sources and transfers them to a suitable format. One of the suitable substrates in the ETL process is the feature manipulation engine (FME), in which a two-way communication is established between IFC and CityGML [50]. FME Reader software, as a converter of IFC information to standard formats in GIS, enables interoperability of data in different formats.

**Fig. 3:**
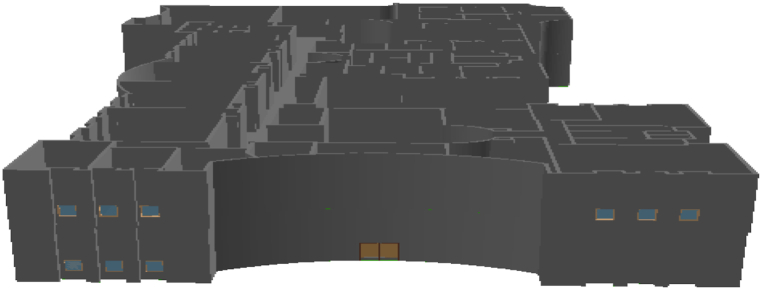
The 3D model of the case study.

In order to provide a 3D model of case study building, Autodesk Revit has been used. In this research, the ground and first floors of the building of the Faculty of Engineering, University of Tehran have been considered as the building case study. First, the 2D CAD map related to the plan of floors was imported into the software and then the 3D process is performed. Walls, ceilings, doors, windows and stairs were considered as the main building elements in providing the 3D model. Then, for each of the mentioned elements, according to the different types in the building in terms of dimensions and type, the specific families related to them are created in the software. In the next step, the drawing of the elements begins; First, the family related to the desired element was selected and then, according to the location of each element on the 2D map of the plan, all the elements related to that family were drawn. This process performed for all families created for different types of elements in the building. After completing the drawing process, the created 3D model can be displayed. Fig. 3 shows the 3D model of the building case study after the 3D modeling process in Revit. Upon completion of the construction of the BIM model of the building case study, the model was converted to IFC format to be used in the next stages of implementation. It should be noted that IFC is an ISO standard and is implemented by many software packages in the building industry. Therefore, it provides a possibility for data sharing, reuse and interoperability.

**Fig. 4:**
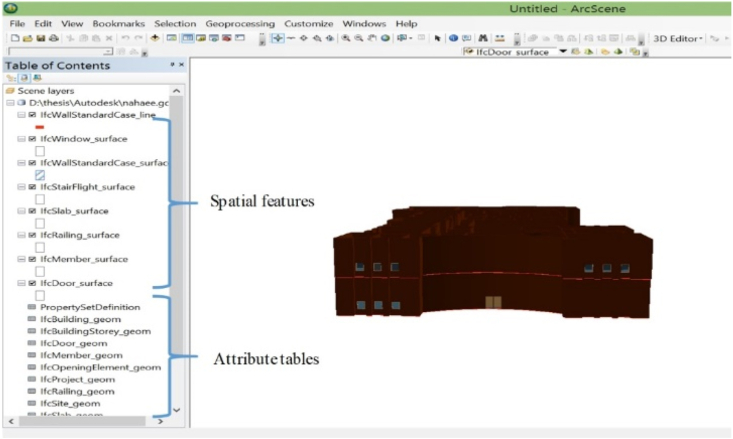
The layers of the IFC model converted to the GDB format in GIS environment.

We used the ArcGIS software package by ESRI for GIS analyses in this research. In order to apply BIM in ArcGIS, the model must be converted to the standard ArcGIS format by means of the Data Interoperability plugin. Firstly, FME Reader software was installed and then was introduced to ArcGIS software. Then, the FME Interoperability tool was activated, and the 3D model of the building was converted from IFC to GDB. All converted data, including tables and 3D layers, were stored in a GDB. Tables contain relationships between layers, and 3D layers contain the geometric properties of the building. Fig. 4 shows the converted layers from IFC format to GDB format in ArcScene software.

#### 4.1.2 Routes network

In order to perform the map-based routing process, two types of spatial models are mostly used: the cell-based and graph-based network [51]. In this study the graph-based network has been exploited due to its simplicity in calculations and the possibility of performing graph-based analyses such as centrality analysis [52]. The main challenge in this research is how to create a 3D route network based on a graph-based model with BIM data that allows the navigation between different levels of the building.

In the proposed approach, the indoor route network is produced by the combined use of the mesh approach and triangulated irregular network (TIN). The Mesh approach focuses only on increasing network accuracy, while the TIN approach includes increasing data utilization efficiency and reducing the difficulty of the network generation process [53].

**Fig. 5:**
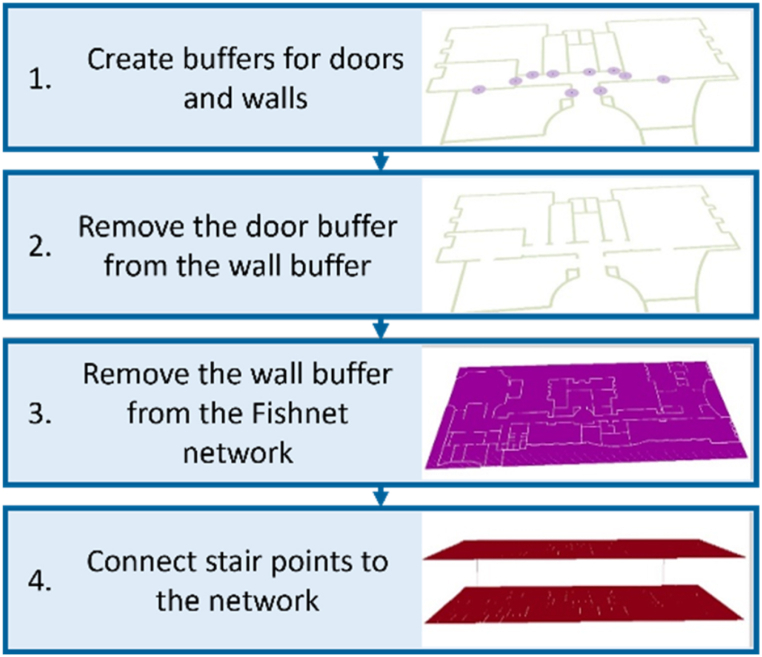
The process of creating the mesh network.

In the mesh method, shown in Fig. 5, the Fishnet tool is initially used to create the network. The intersections between the walls and the network are removed to create a feasible routing network. To do this, the door layer is converted to a point layer and, based on the door and wall widths, the two respective buffers are created (Fig. 5, step 1). Next, the door layer buffer is removed from the wall layer buffer using the Erase tool (Fig. 5, step 2). At this point, this area is removed from the Fishnet network because the wall area should not interfere with the network (Fig. 5, step 3). Finally, to allow the connection between the different levels of the building, the final path network is constructed by connecting the starting and ending points of the stairs of the different levels to the network with a line (Fig. 5, step 4).

**Fig. 6:**
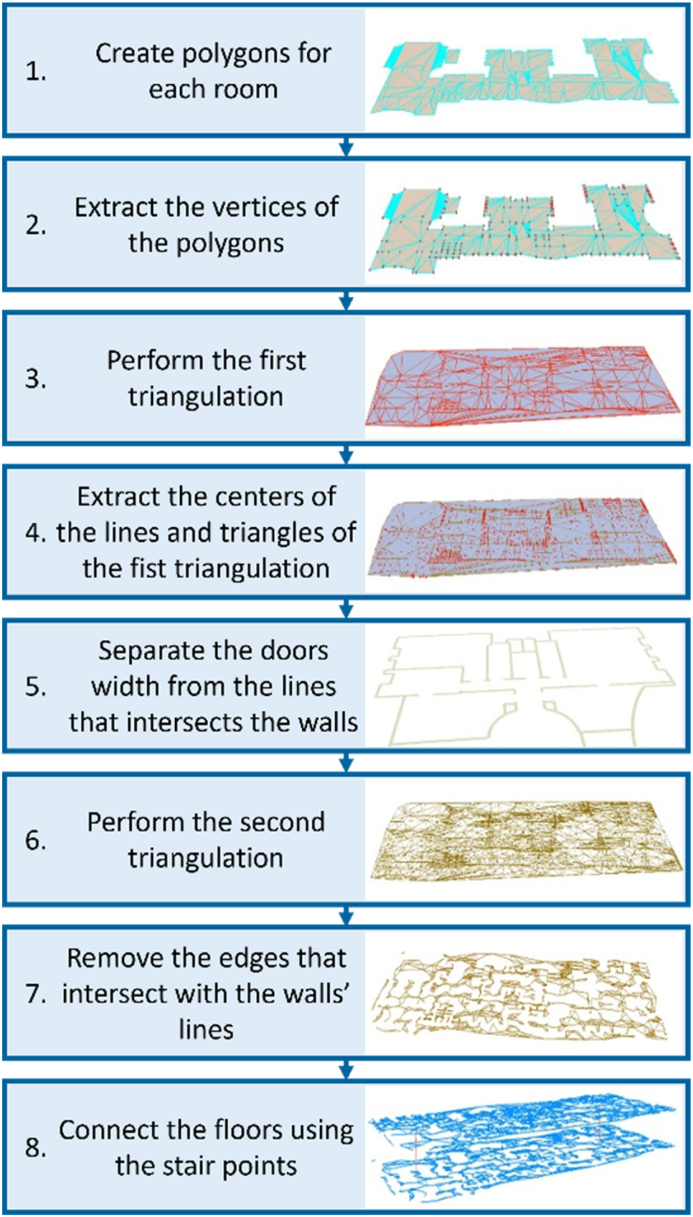
Creation of the final route network by TIN method.

The TIN method, shown in Fig. 6, was used to create the route network by triangulation. This method receives the wall level as input and initially creates a polygon for each room (Step 1 in Fig. 6). Since walls are barriers in the route network, the points used as input for the triangulation are obtained by extracting the vertices of the wall lines (Step 2 in Fig. 6). These points were then used with the door points to perform the first triangulation The door points were included because the doors should be considered as part of the path network. (Step 3 in Fig. 6). The set of route points is obtained by extracting the center points of the triangulation lines (Step 4 in Fig. 6). Then, the width of the doors must be removed from the edges that form the wall (Step 5 in Fig. 6). A second triangulation is performed to create the edges of the route network (Step 6 in Fig. 6). To obtain only feasible paths, the edges that intersect with the wall lines are removed (Step 7 in Fig. 6). Finally, ladder points were used to connect the planes together, and the final path network was completed (Step 8 in Fig. 6).

Finally, the created route network was shared as a web service allowing to be used at any time. There are several software packages available to provide the route network as a web service, including ArcGIS server, GeoServer, etc. In the present study, the ArcGIS Server was used to establish the web service required.

#### 4.1.3 Indoor positioning

**Fig. 7:**
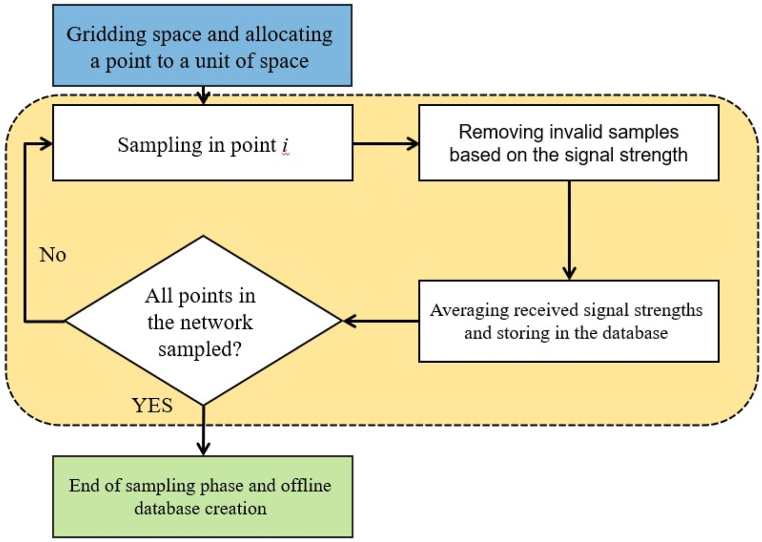
The flowchart of offline database creation.

**Fig. 8:**
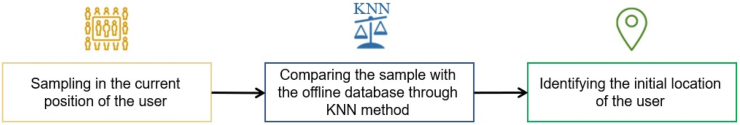
The general steps of driving user's initial position.

Regarding the initial position of the user, the fingerprinting method was used. In order to implement the fingerprinting method, an offline database is required. Fig. 7 shows the process for creating the offline fingerprinting database through sampling all points in the network created and Fig. 8 illustrates the general steps for obtaining user's initial location from sampling signals in the user's initial position and then by comparing the sampling result with the database the location is acquired.

**Fig. 9:**
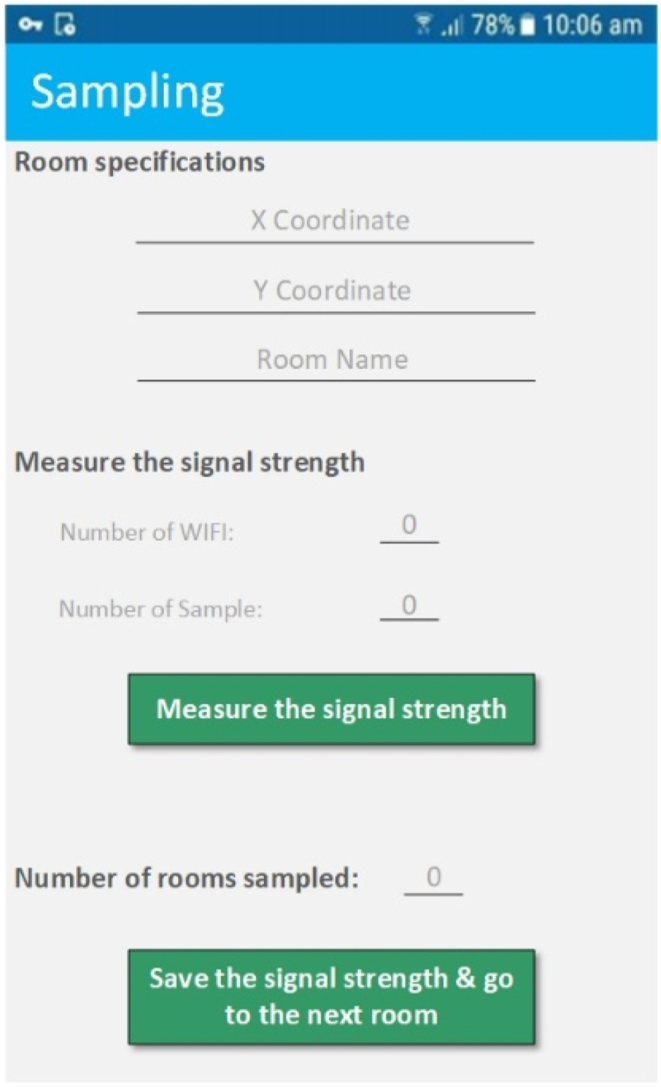
The user interface of the system.

In this research, for automating and simplifying the sampling phase, a mobile app was developed. Within the app, the concepts of JSON array and JSON object have been used to create an offline database. One of the advantages of this type of data storage over relational databases, such as SQLITE is their non-structural nature. This feature helps us to be able to store all the Wi-Fi endpoints that can be viewed anywhere and use them in the online phase. Table 1 shows JSON arrays used. The user interface (UI) implemented to create the offline database is presented in Fig. 9.

**Table 1:** The JSON arrays used in this research.

JSON array name	Description
AllSamples	Saves all Wi-Fis that are found along with their BSSID and RSSI for n times to calculate signal strength at one point.
FinalTestArray	Saves cumulative BSSID and RSSI values along with the number of repetitions of all unique Wi-Fis found in which RSSI values are outside the designated RSSI range of each BSSID are removed.
FinalMean	Saves the average BSSID and RSSI values along with the number of repetitions of all unique Wi-Fis found in which the RSSI values outside the designated RSSI range of each BSSID are removed. Also stored here are information values for each point (such as X and Y and room number).

According to the figure, by standing in each room or any part of the corridor, we first find the X and Y coordinates of the desired point from the map created in the previous section and enter them in the specified parts. In addition, to separate the different rooms from each other, a room name must be specified at each point. It should be noted that this room name must be unique so that it can be used in later stages. After entering these values in the signal strength measurement section by clicking on the signal strength measurement option, the signal strength of all access points (APs) found at that point along with their basic service set identifier (BSSID) is stored in the *AllSamples*. At this stage, the user can measure the signal strength of APs as many times preferred. After measuring the signal strength in the desired room by clicking on the option to save the signal strength, first for the signal strength of the unique BSSIDs found, we consider a minimum and maximum range according to the measured values for that BSSID to reduce errors that may occur in measuring the strength of the BSSID signal. Then for each of the BSSIDs at this point we calculate the sum of the signal strength values that are in the specified range. We store these values in the *FinalTestArray*. Finally, after calculating these values, we store the average of the total values of the signal strengths along with the room specifications in *FinalMean*. After completing these steps, we go to the next room, with *AllSamples* and *FinalTestArray* empty. At the end of the sampling step, the value of *FinalMean* is the output of this step, which in the online phase will be used to find the user's initial location.

**Fig. 10:**
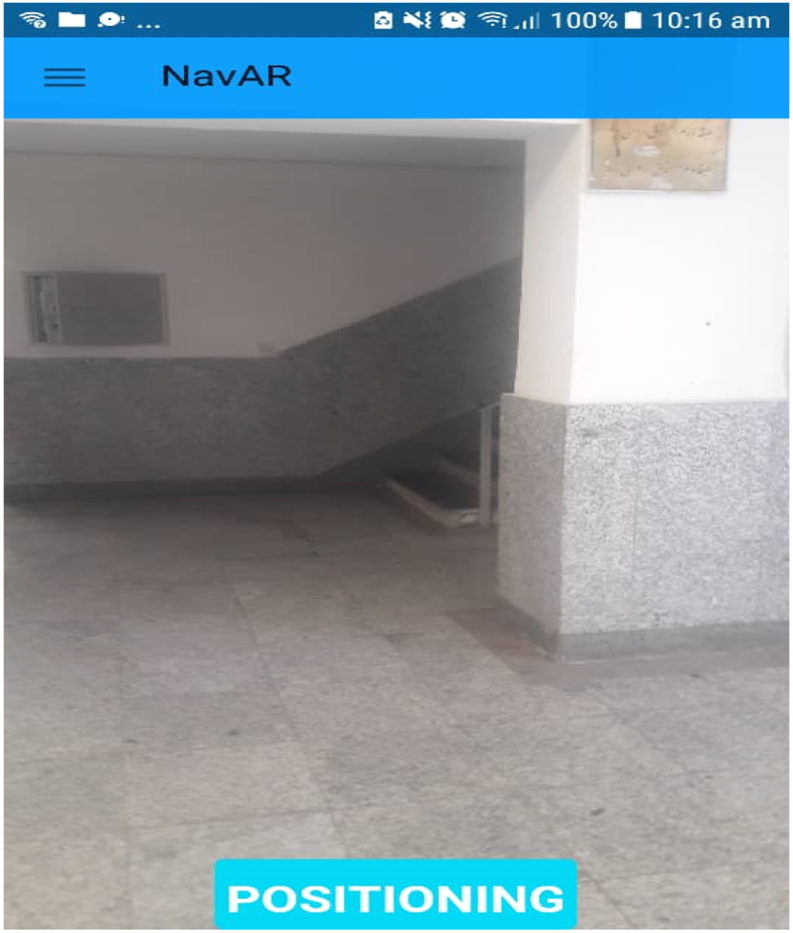
Obtaining initial position in online mode through the offline database.

In order to find the user's position online, the KNN method was used to compare the *FinalMean* array online with the created offline database. Actually, according to Fig. 10 in the online mode, by clicking on the *POSITIONIG* option, we first use the Receiver Wi-fi class to store all the APs that are currently available, along with the signal strength of each of them, in an array called *allSamplesForPositioning*. Through Equation (1), we obtain the signal strength difference of each of the APs available online with the offline database created. It should be noted that the weakest signal strength value is assigned for APs that do not have a signal strength value in the sample.

(1) $$D_{j}=\sqrt{\sum_{i=1}^{m}{\left(s_{i}-s_{ij}\right)}^{2}}$$

where $D_{j}$ is the distance from the sample j, m is the number of APs, $s_{i}$ is the signal strength received from the i ^th^ AP in the online phase, and $s_{ij}$ is the signal strength of the i ^th^ AP stored in the j ^th^ sample.

In recent years, many methods are presented based on local fingerprinting and Wi-Fi to determine the location in indoor environments. Each of these methods uses a different positioning algorithm and has different performance. Table 2 examines the types of these methods and the efficiency of each of the different perspectives and compares them with the method used in this research.

**Table 2:** The comparison of various approaches of fingerprinting indoor positioning.

Wireless technology	Positioning algorithm	Accuracy	Precision	Complexity	Scalability	Robustness	Cost	Reference
WLAN RSS	KNN	1.5–10 m	80 % within a radius of 1 m	Low - high	Good	Good	Low	[54,55]
WLAN RSS	KNN + Clustering	1–3 m	90 % within a radius of 5 m	Medium - high	Good	Good	Low	[56]
WLAN RSS	Probabilistic Methods	1.5–3 m	80 % within a radius of 2 m	Medium - high	Good	Good	Low	[57]
WLAN RSS	ANN	1.5–3 m	80 % within a radius of 2.5 m	Low	Poor	Good	High	[57,58]
WLAN RSS	DL	0.5–3 m	75 % within a radius of 1.5 m	High	Medium	Good	High	[59,60]
WLAN RSS	KNN	1.5–3 m	85 % within a radius of 5.2 m	Low	Good	Good	Low	Proposed approach

As it can be seen, the accuracy of the RSS WLAN method for this research is 85 %. This accuracy was obtained using 42 samples. For this purpose, in the online phase, the local fingerprinting method is used to calculate the user's position in different areas of the building. The method correctly identified 36 samples. As mentioned earlier, the first step is to know which room or area the user is in. The method used performs this operation correctly and with high accuracy. One of the most similar methods to the one used in this research is the hallway-based method presented in Ref. [61]. In this method, the purpose was to partition the space, in other words, to determine the room or user area. This method only used RSS WLAN. Furthermore, the accuracy of the positioning was 92 %. According to Table 2 and what was mentioned previously, the method used in this research is appropriate to the purpose of the research and has the necessary efficiency. Low complexity for implementation, acceptable stability and robustness in various places and times and sufficient accuracy for determining the initial position of the user are among its features.

After obtaining the initial position of the user, in order to find the exit route from the building, we use a web service published on ArcGIS Server, which is described in Section IV.A.2. In this research, it is assumed that the exit route from the building is only one point. Therefore, the main objective here is to find the shortest path between the two points. This action is performed by the ArcGIS software that implements the Dijkstra algorithm. Due to the relatively small network, this algorithm has been considered more flexible than A* because it does not require a heuristic distance function.

**Fig. 11:**
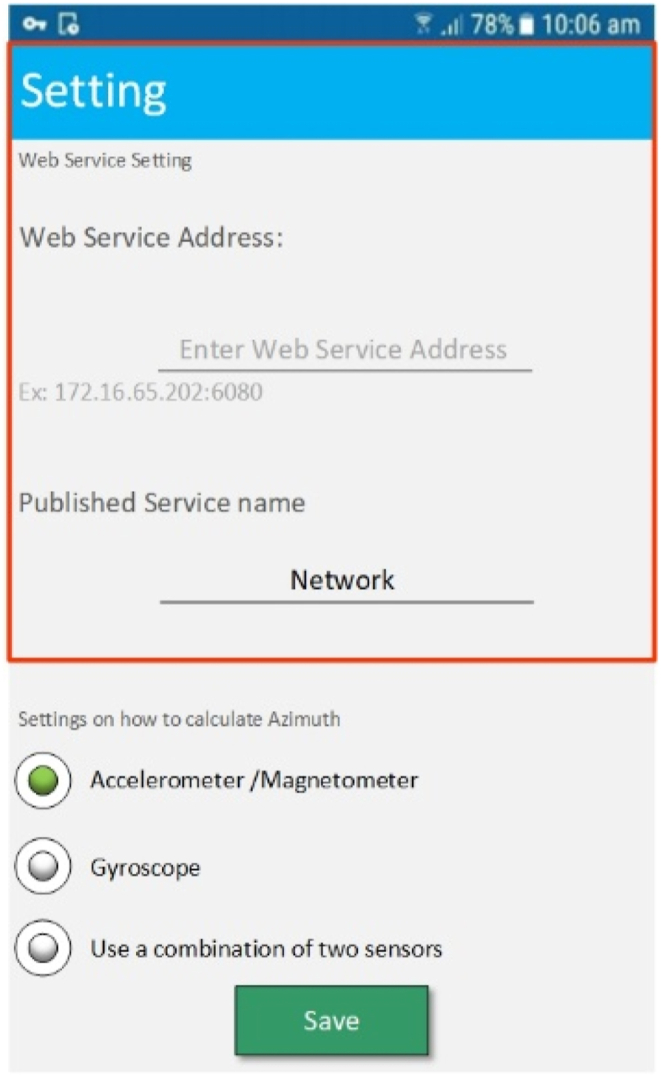
The URL and service name in setting up of the application.

At this stage, the client and the server must be connected to a local network so that they can communicate with each other. Therefore, the first step in implementing this section is to introduce the web service address and the name of the web service to the program. This can be done, as shown in Fig. 11, in the program settings section. It should be noted that by setting this address, using a concept called shared preference; this address can be used in other classes and activities of the program.

The most important function for this web service is the “*Solve*” function. This function can be implemented on route network service. The most significant inputs of this function, along with descriptions of each, can be found in Table 3.

**Table 3:** The parameters of the solve function.

Parameter	Description
F	Defines the format of the output. The default is HTML. Values: HTML/JSON
stop	The first and last points are determined in this part. A sample is shown in JSON in the following figure.
	{“ type”: “features”,
	“hasZ”: <true|false>,
	“features”: [
	{“ geometry”: {<geometry1 >},
	“attributes”: {“<field1 >”:
	<value11 >, “<field0>”: <value10>}
	},
	{“ geometry”: {<geometry0>},
	“attributes”: {“<field1 >”:
	<value01 >, “<field0>”: <value00>}
	}
	]}

#### 4.1.4 User navigation

**Fig. 12:**
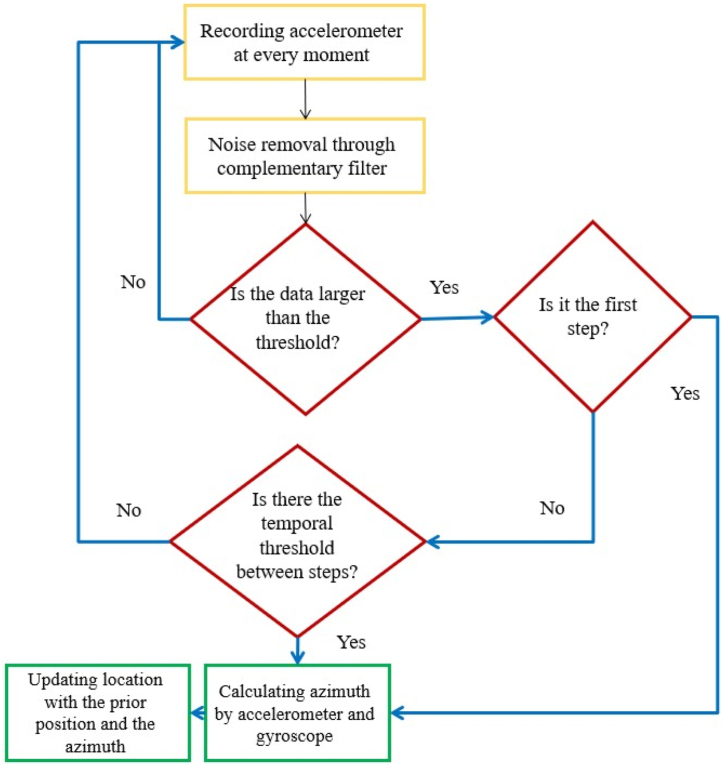
The flowchart shows the process of continuously updating the user's position.

After taking the initial position of the user, this position (y, x) is sent to the server, and according to what was described in the previous section, the exit route of the building from the desired point is determined by the map service created in ArcGIS Server and the path is returned to the client. After receiving the output path, the user's position will change as the user moves towards the exit point. Therefore, it is necessary to update the user's position at any time. Fig. 12 shows the flowchart of the implementation steps to update the user's position.

**Fig. 13:**
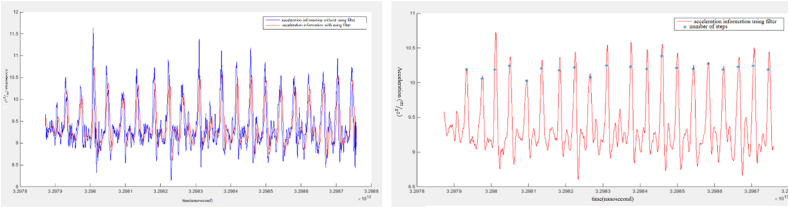
The acceleration sensor information for 20 steps and the results of the number of steps.

In this regard, pedestrian dead reckoning (PDR) method was used to update the user's position. The calculation of the number of steps while the user is walking can be obtained by using the information related to the accelerometer sensor. In order to increase the accuracy of the information related to acceleration and eliminate potential noises, the moving average filter has been used. There are three major approaches for step detection in PDR: 1) zero crossing, 2) peak detection and 3) flat zone identification. In this study, the simple peak detection [55] was employed. Fig. 13 shows the acceleration sensor information for 20 steps using and without using the filter. As you can see in the figure, the number of peak points in the diagram indicates the number of steps. The Figure also shows the results for the number of steps calculated. As it can be seen, the 20 steps for the user's movement are detected correctly.

After recording each step, according to the previous position of the user, its current position will be obtained. In this research, the step length for the user is considered 0.7 m [62]. Obtaining the user's position in PDR approach is computed according to Equation (2), which requires azimuth or user orientation.

(2) $$\left\{\begin{array}{c} x_{i}=x_{0}+\sum_{i=1}^{n}d_{i}cos\theta_{i}\\ y_{i}=y_{0}+\sum_{i=1}^{n}d_{i}\;\sin\;\theta_{i} \end{array}\right\}$$

where ($x_{i}$, $y_{i}$) is the current location of the user and ($x_{0}$, $y_{0}$) is the previous location of the user. *d* is the distance between two locations that the user has moved (step length) and *theta* is the azimuth or bearing angle of the user's direction. For acquiring the bearing angle, we have used the information of the smartphone sensors, which is discussed in detail in the next section.

### 4.2 AR interface

#### 4.2.1 Direction calculation

**Fig. 14:**
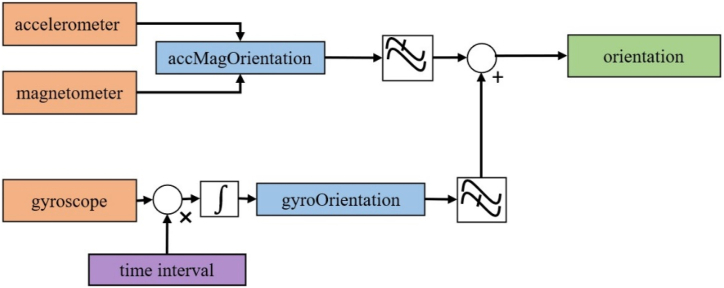
The complementary filter used to refine position estimation.

In order to calculate the azimuth in this research, the combination of smartphone sensors with a complementary filter was used. A simple complementary filter described in Ref. [63] can yield good results. In this method, as in Fig. 14, a low-pass filter is used to process the data from the accelerometer and compass. This filter by averaging a certain number of measurements makes the output signals smoother. On the other hand, high-pass filter can act as an input gate, in which only values that have changed sufficiently from the previous value are allowed. Since the gyroscope can only measure angular velocities, therefore, there is a need for an integration step to reach the direction of the gyroscope data. During this integration, the existing noise is converted into drift. This drift error can be eliminated through a high-pass filter. The figure shows the structure of a complementary filter that integrates the information of accelerometer, magnetometer and gyroscope sensors to estimate the absolute orientation of the device without drift error.

As shown in Fig. 11, there are three ways that the application can compute the direction from which the user can choose: the accelerometer/magnetometer, the gyroscope or a combination of both. In order to evaluate the accuracy of location updating using these methods, Equation (3) has been used. Table 4 compares the results of the location estimation accuracy of the three approaches discussed above, which are obtained in five distance intervals.

(3) $$\delta_{xy}=\sqrt{\delta_{\begin{array}{c} x \end{array}}^{2}+\delta_{\begin{array}{c} y \end{array}}^{2}}$$

**Table 4:** The comparison of the different azimuth calculation methods.

Distance	Azimuth calculation method		
	Accelerometer/magnetometer	Gyroscope	hybrid
20 m	0.56 m	0.3 m	**0.17 m**
40 m	0.73 m	0.62 m	**0.27 m**
60 m	0.82 m	0.78 m	**0.45 m**
80 m	0.89 m	0.92 m	**0.52 m**
100 m	1.05 m	1.25 m	**0.78 m**

#### 4.2.2 Path visualization in AR

**Fig. 15:**
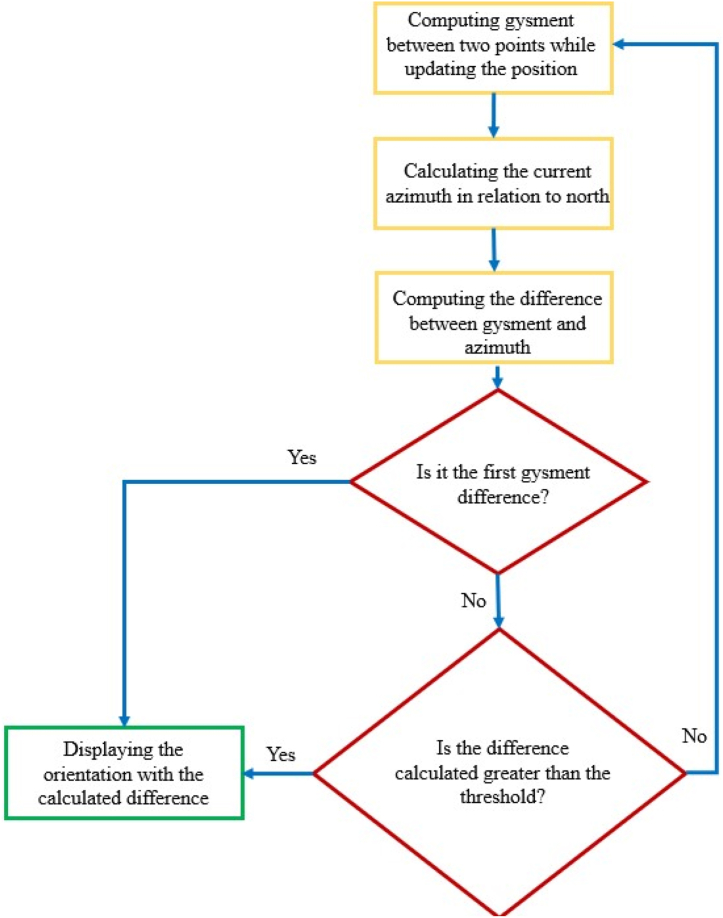
The flowchart of direction visualization.

The mobile AR visualization is used to show the exit path to the user. Fig. 15 presents the flowchart of the process that displays the right direction to the user.

**Fig. 16:**
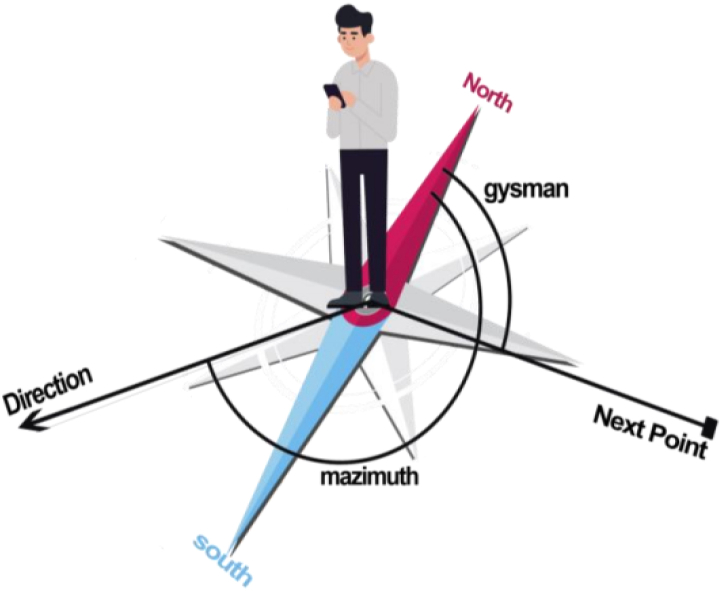
The updating of the direction shown to the user.

According to Fig. 16, the *mAzimuth* angle will alter as the direction of the user's movement changes. Therefore, after the changing of that angle, the angle shown in the smartphone screen (*ShowGysman*) ought to change, which is computed using Equation (4). Given that azimuth detection sensors are very sensitive and they record small changes, a threshold should be used for the angle changes obtained in Equation (5), otherwise the direction shown to the user will change at any time and the program will lose its functionality. It should be noted that this threshold has been obtained empirically.

(4) ShowGysman=−|Gysman–mAzimuth|

Given that azimuth detection sensors are very sensitive and they record small changes, a threshold should be used for the angle changes obtained in Equation (5), otherwise the direction shown to the user will change at any time and the program will lose its functionality. It should be noted that this threshold has been obtained empirically.

(5) TR=−|ShowGysman–OldShowGysman|>20°

**Fig. 17:**
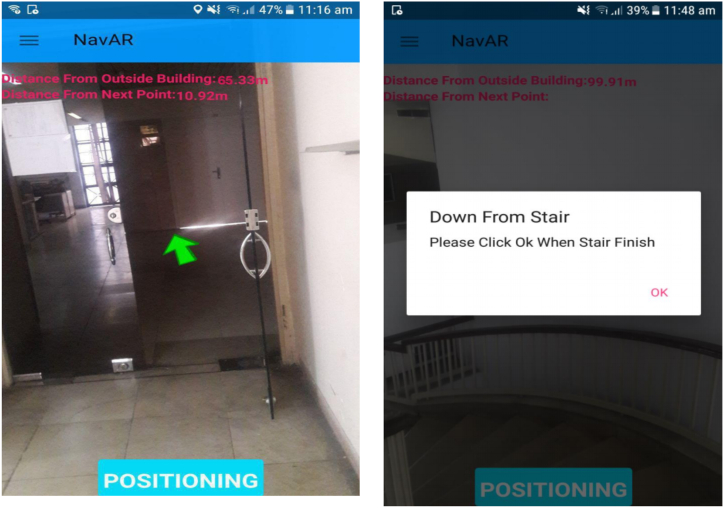
The navigational information superimposed on the smartphone screen.

Also, additional information such as the distance to the next node and also the distance to the exit, is displayed to the user at any time. As previously described, the stairs are modeled as a straight line that connects the beginning and ending points of the stairs. Therefore, using the proposed method, the user cannot be tracked when crossing the stairs. To solve this issue, the concept of AlertDialog has been exploited in Android programming. In fact, when the user enters the stairs, a message is displayed, and when reached the end of stairs, the user notifies the program by pressing a button. Fig. 17 shows exit path visualization to navigate the user and the message displayed to the user while reaching the stairs.

## 5 Evaluation

To evaluate the mobile AR application developed in this research, the system usability scale (SUS) test [64] has been used. The SUS test is a questionnaire that is used to measure the usability of a system. The SUS system calculates the system usability through a survey after a user uses the system. While SUS is used today to measure the usability of websites, its use is not limited to the website, but the system can be used to measure any system and applications of digital products such as mobile applications [65].

This questionnaire (Table 5, in Appendix A) designed had 10 questions with 5 scores. All the questions are centered around the usability of the system developed. Each question can be rated from 0 to 4, with 0 meaning strongly disagree and 4 meaning strongly agree. The questions with odd numbers (1-3-5-7-9) had a positive aspect of the system and questions with even numbers (2-4-6-8-10) had a negative aspect towards the system. The scoring method of this questionnaire is such that the score of each odd question minus one, and for the even questions, the value of 5 minus the score of that question determines the number of points for that question. Finally, total sum of points of the questions will be multiplied by 2.5. The mathematical expression of the formula is presented in Equation (6). In the best case, the score will be 100 and the worst score will be 0. During research on 511 studies, a SUS test score above 68 is considered above average, and any score below 68 is below average [64,65].

(6) SUSScore=((Q1−1)+(5−Q2)+(Q3−1)+(5−Q4)+(Q5−1)+(5−Q6)+(Q7−1)+(5−Q8)+(Q9−1)+(5−Q10))*2.5

**Fig. 18:**
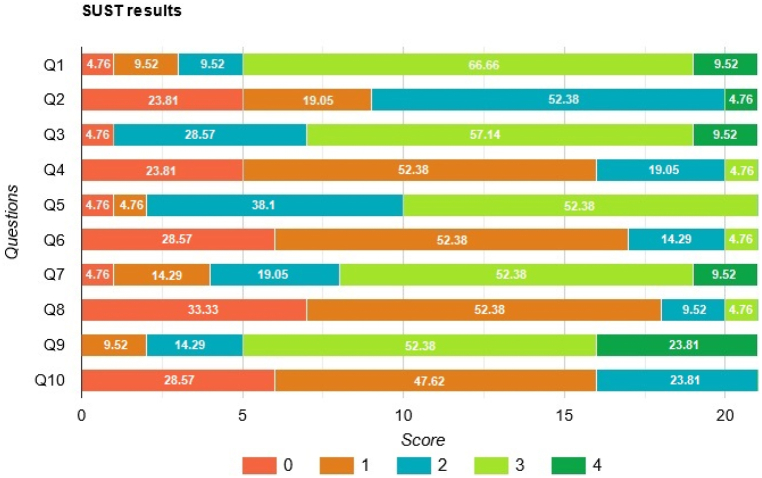
The SUS test user evaluation results.

We used individuals both familiar and unfamiliar to the test area. All participants expressed their consent to take part in the usability test. The statistical sample of this research was a total of 21 people, 10 (47.6 %) of whom were familiar with the environment and 11 (52.4 %) of them were unfamiliar with the environment. Also, the random stratified sampling method was used to ensure the sample reflects a close-to-real representation of the people in the building. The sample size was chosen based on studies in a relatively similar scope; in addition, some other researchers in recent studies have found that a number of participants around 15 is enough for assessing mobile applications [66]. Fig. 18 shows the results of user evaluation and the percentage of scores for each question. As it can be seen, in all of the odd questions at least more than 50 percent of users agree or completely agree to the efficacy of the system and the opposite is true for the even questions. The SUS test score of this study is 70.59, which shows that the users evaluated the usability of the application as above average.

**Fig. 19:**
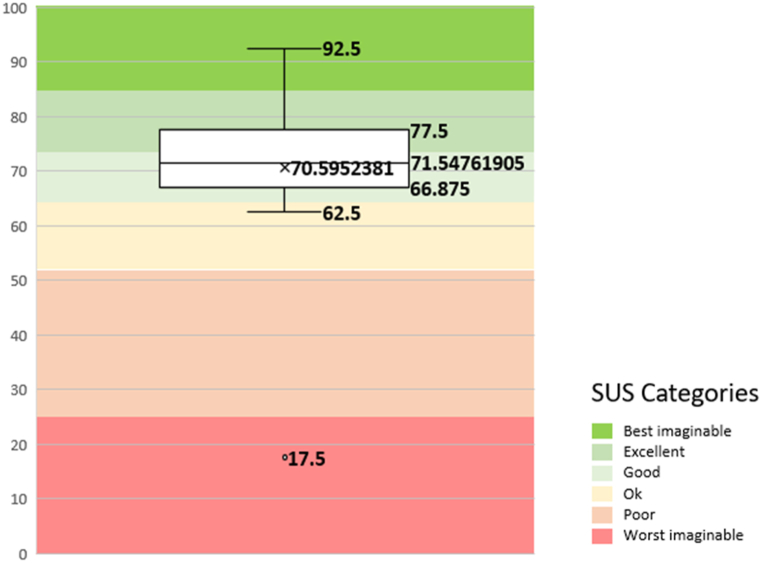
The boxplot of the SUS test evaluation scores.

The SUS evaluation scores were also plotted as a boxplot chart in Fig. 19. The SUS score can be divided into six categories as follows: worst imaginable (0–25), poor (25.1–51.6), OK (51.7–62.6), good (62.7–72.5), excellent (72.6–84.0), and best imaginable (84–100) [66]. As it can be seen, the mean SUS score is in the good category range.

## 6 Conclusion

Recently, research has shown more and more interest in emergency evacuation of buildings since this is an integral part of urban environment management. In this research, we proposed an end-to-end approach by utilizing the capabilities of BIM model. Then, by converting it to the GIS environment we could create a network for indoor space. Moreover, a mobile app was developed that in first phase created an offline database of different areas in the building through the fingerprinting method. In the online phase this app calculated the user's initial position by comparing the received signal strengths from the APs around to the offline database. Then, the exit route was computed, and the user was navigated in the indoor environment by taking advantage of the smartphone built-in sensors. Furthermore, AR was used to convey the path direction to the user. Eventually, the proposed method was evaluated with a sample audience that achieved acceptable results with a SUS score of 70.59.

However, there were numerous limitations involved in this research. The most important point to note is that only one exit point was considered. In real life situations, usually there are multiple exit and emergency exit points in buildings; in addition, also windows of the first floor can be in some cases regarded as exits. Future studies can take into consideration all these exits and could consider tant some of these exits might not be accessible due to fire or debris blocking. Another point to focus is the crowd congestion in exits that should be thought out for an optimal evacuation. Furthermore, designing various scenarios for a better modeling of the emergency evacuation is necessary. In addition, incorporating alert systems for dynamic monitoring of hazard spread can be very much beneficial.

An issue that needs further investigation is WLAN sensors network. Accuracy of Wi-Fi positioning can vary a lot. This is partly due to the placement of the Wi-Fi sensors in the building environment which can affect RSSs of the sensors and the coverage of all indoor space with presence of signals from sufficient number of sensors. As a result, optimized placement of sensors can increase the accuracy of the positioning, which is extensively studied by Hosseini et al. [67]. Also, future works can focus on studying the effects of addition or removal of access points and network latency on the positioning accuracy. In order to increase the system robustness, there should be measures taken while certain aspects that system relies on is not available. For example, in case of WLAN sensors not working the system will not be able to locate the start point of the user to compute the path. However, since the system only requires the Wi-Fi sensors for initial positioning of the user, it can be said that the degree of reliance on WLAN sensors is not high, and this can be solved by providing an option for the user to indicate the name of the nearest room for the system to identify a close estimation of the user's initial location. From user-friendliness point of view, the visualization of the exit path requires implementation of elements that are more close to human wayfinding.

For improving this research, there are a number of suggestions for future studies. The newer versions of ESRI ArcGIS Pro have incorporated FME reader, which offers easier handling of the Revit 3D models. The possibility of direct usage of 3D models within GIS environment can be an interesting area for further studies. Another area of improvement could be employment of artificial intelligence (AI) in the positioning phase, especially with the recent improvements in fast and less complex algorithms. Additionally, other means of path visualization and navigation techniques, such as virtual reality (VR) and mixed reality (MXR) along AR should be explored in regard to user preference, behavior and wayfinding performance. It can be more beneficial if it is combined with user profiling (age, sex, presence of disabilities, etc.) to provide a more context-aware guide. Another useful idea is to take advantage of spatial features of the building that are familiar to its inhabitants [68,69]. This is more aligned to human mind in path finding and navigation. Therefore, ideas such as indoor tagging of spatial features and sematic annotation of specific points of interest (POI) in the BIM model of the building can be beneficial [70]. This can provide further insight in terms of system effectiveness and limitations of the system to better decide on the suitable architecture for a more useful evacuation system. Finally, in terms of better evaluation of user experience (UX) and usability of the system, we propose to include more user samples along with developing some hypotheses regarding the correlation of the trend of user rating by other influencing factors, such as age and familiarity with the location and AR apps to test them with more comprehensive statistical approaches.

## Data availability

The codes and data are available upon request from the authors.

## CRediT authorship contribution statement

**Mojtaba Valizadeh:** Writing – original draft, Visualization, Methodology, Investigation, Data curation, Conceptualization. **Babak Ranjgar:** Writing – review & editing, Writing – original draft, Visualization, Validation, Supervision, Project administration, Methodology, Formal analysis. **Alessandro Niccolai:** Writing – review & editing, Supervision, Formal analysis. **Hamid Hosseini:** Writing – original draft, Visualization. **Soheil Rezaee:** Visualization. **Farshad Hakimpour:** Writing – review & editing, Supervision.

## Declaration of competing interest

The authors declare that they have no known competing financial interests or personal relationships that could have appeared to influence the work reported in this paper.

## Figures and Tables

**Table 5:**
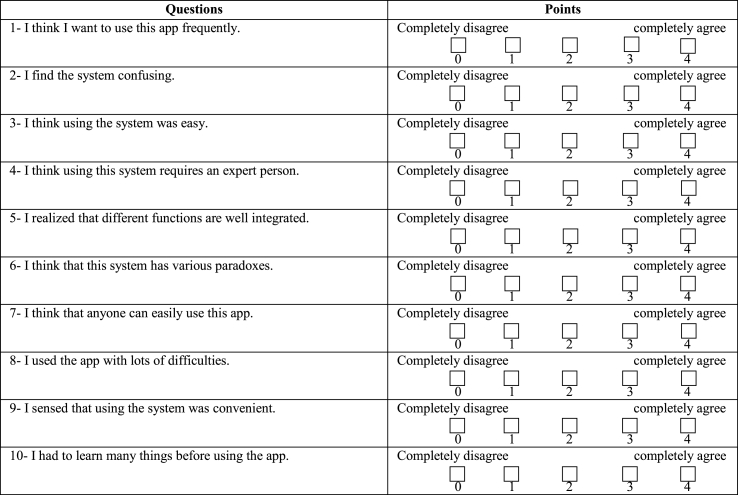
The questionnaire of system usability.
